# Risk factors for postoperative acute kidney injury in overweight patients with acute type A aortic dissection

**DOI:** 10.1186/s13019-023-02218-0

**Published:** 2023-04-08

**Authors:** Yu Xu, Shang-Tai Dai, Lin-Zhuo Liu, De-Mei Kong, Shi-Kui Guo, Kun-Mei Gong

**Affiliations:** Affiliated Hospital of Kunming University of Science and Technology/The First People’s Hospital of Yunnan Province, Kunming, 650000 Yunnan China

**Keywords:** Acute kidney injury, Acute type a aortic dissection, Risk factors

## Abstract

**Objective:**

To analyze the clinical characteristics of patients with overweight acute type A aortic dissection, and to explore the risk factors of acute kidney injury in patients with overweight acute type A aortic dissection.

**Methods:**

From March 2019 to February 2022, the clinical data of 71 patients with acute type a aortic dissection diagnosed by CTA and undergoing surgical treatment with BMI > 24 in the First People's Hospital of Yunnan Province were retrospectively analyzed, and analyzed by univariate and logistic multivariate analysis methods.

**Results:**

The mean BMI of all included patients was 27.23, The mean surface area of all included human populations was 1.833. The mean age of all patients was (52.06 ± 10.71) years old, and 35 patients developed acute kidney injury after surgery. Multi-factor Logistics regression analysis confirmed the risk factors for postoperative acute kidney injury in overweight patients with acute type A aortic dissection, including gender, CPB transit time and intraoperative infusion of suspended red blood cells. Seven patients in the AKI group died in hospital after surgery and two patients died in the non-AKI group.

**Conclusions:**

Among patients with overweight acute Type A aortic dissection, the incidence of AKI is 49.30%. According to multi-factor Logistics regression analysis, gender, CPB transit time and intraoperative suspended red blood cell volume are independent risk factors for postoperative acute kidney injury in patients with overweight acute Type A aortic dissection.

## Background

Acute type a aortic dissection (ATAAD) is a serious life-threatening cardiovascular emergency that increases mortality by 1% to 2% per hour, The incidence is 2.6 to 3.5 per 100,000 every year in china [1], At the same time, misdiagnosis often delays the patient's condition, which should not be ignored. Therefore, emergency CTA examination is necessary for patients with sudden chest and back pain, and emergency surgery is the only way to save the patient's life [2]. Acute kidney injury (AKI) is a common postoperative complication of ATAAD patients [3], and also a common risk factor of nosocomial death in ATAAD patients [4]. Related studies have confirmed that the occurrence of postoperative AKI significantly increases the mortality of patients [5]. Many studies have reported the mortality and related risk factors of AKI after ATAAD [6]. Overall, the risk of develping AKI and the proportion of patients dying from AKI is higher. At the same time, obesity has been identified as a risk factor for heart-related surgery [7], which can increase the risk of surgery for ATAAD patients [8].

Some reports suggest that AKI is more likely to occur in overweight ATAAD patients [9]. Body mass index (BMI) is positively correlated with a higher incidence of nosocomial serious adverse outcomes in patients undergoing ATAAD surgery [10]. However, there are few reports on risk factors for postoperative AKI in overweight ATAAD patients. The purpose of this study was to investigate the risk factors for postoperative AKI in overweight ATAAD patients.

## Materials

### Study population

**Fig. 1:**
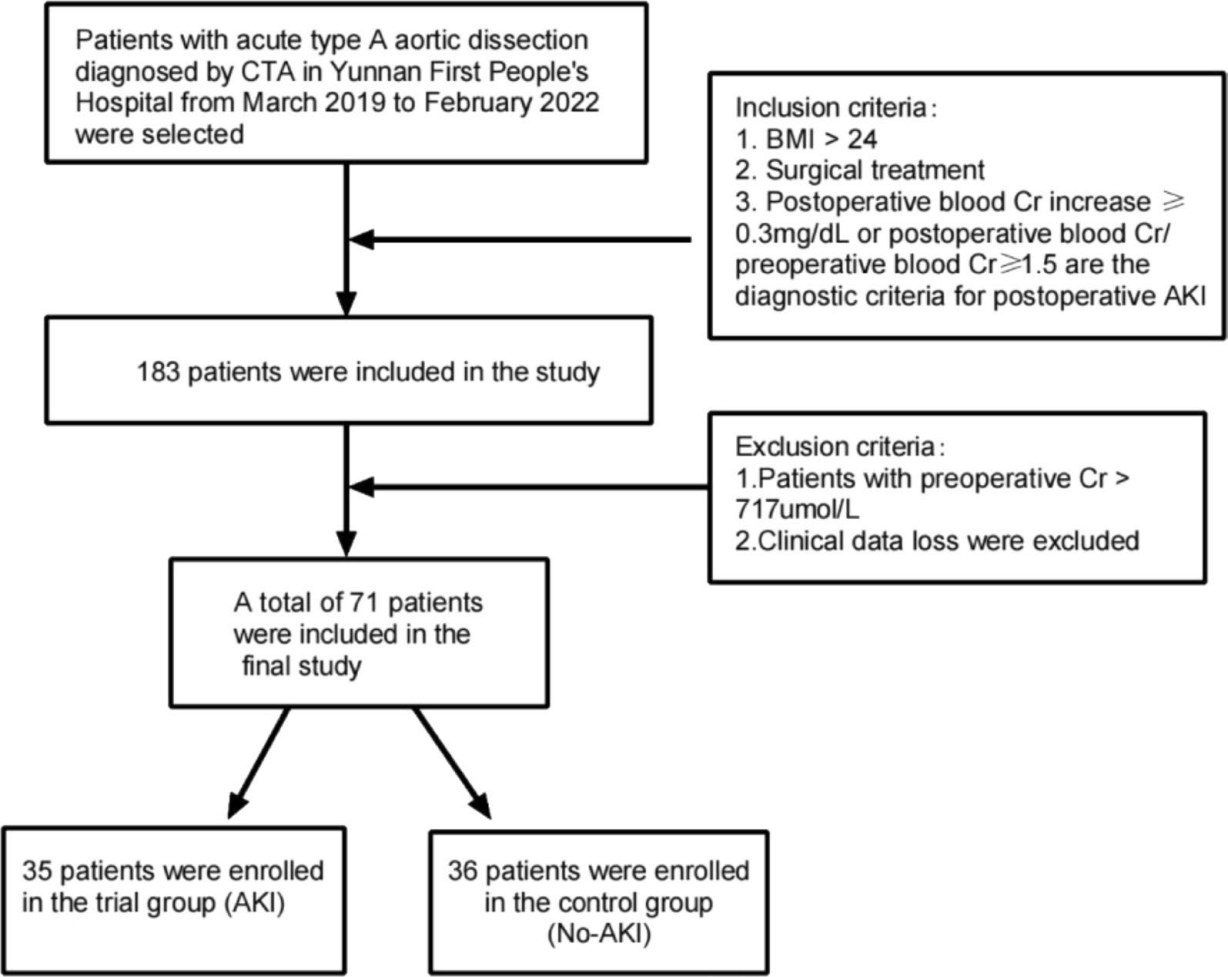
Flow chart of the study

This study retrospectively analyzed the clinical data of patients with acute type A aortic dissection diagnosed by CTA and with BMI > 24 who underwent surgical treatment in the First People's Hospital of Yunnan Province from March 2019 to February 2022. Patients with preoperative Cr > 717umol/L and clinical data loss were excluded, and 71 patients were included in this retrospective study (Fig. 1).

### Diagnostic criteria for postoperative AKI

**Fig. 2:**
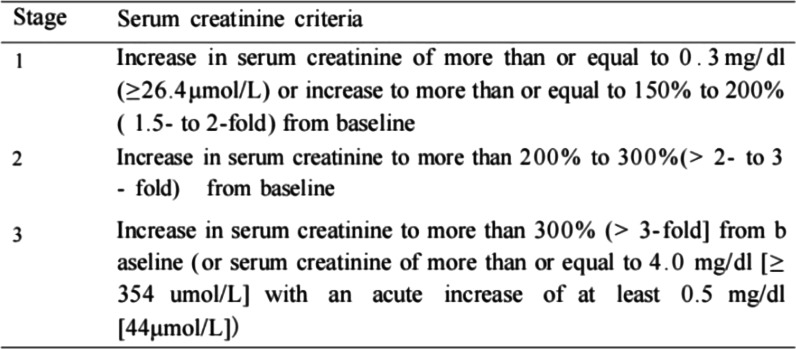
Diagnostic criteria for postoperative acute kidney injury

The diagnostic criteria for postoperative AKI in this study were based on the 2007 Acute Kidney Injury Network (AKIN) [11] (Fig. 2). Postoperative blood Cr increase ≥ 0.3 mg/dL or postoperative blood Cr/ preoperative blood Cr ≥ 1.5 are the diagnostic criteria for postoperative AKI.

### Surgical procedure and postoperative treatment

All patients experienced Sun’s surgery, Femoral and axillary arteries were separated for arterial cannulation After routine anesthesia, endotracheal intubation, disinfection and towel laying. The ascending aorta, aortic arch, innominate artery, left common carotid artery, left subclavian artery and descending aorta were gradually dissociated. In the condition of systemic heparinization, reduce the nasal temperature to 25 degrees Celsius and anal temperature to 29 degrees Celsius, total aortic arch replacement and elephant trunk stent placement were performed after the establishment of artificial cardiopulmonary bypass, Stepwise vascular anastomosis (anastomosis sequence: proximal descending aorta, left common carotid artery, left subclavian artery, innominate artery after stent release, and ascending aorta after reperfusion), It can reduce ischemia to the brain and various organs as much as possible, and restore blood supply. Finally, the operation was completed after cardiac resuscitation, weaning and hemostasis. All postoperative patients with endotracheal intubation for treatment in the ICU, as far as possible using corresponding drugs for postoperative organ function support, help the recovery of patients with organ function, and finally pulled out endotracheal intubation, step by step to reduce vascular active drug dose, the functional recovery of patients with organ eventually reached the standard of the ICU, until discharged from the hospital.

### Statistical methods

For the statistical analysis, this study adopts the spss20.0 to normality test of continuous variables, in accordance with normal distribution using t test, does not conform to the normal distribution of the rank and inspection, classification variables using chi-square test, has the significant variables in the single factor analysis into the multi-factor Logistic regression analysis, for all of the analysis, Probability value (P) less than 0.05 is considered statistically significant.

## Results

The mean BMI of all included patients was 27.23. The mean surface area of all included human populations was 1.833. Table 1 shows the basic clinical data of this study. A total of 71 patients were included in this study, including 49 males and 22 females. The average age of the included patients was (52.06 ± 10.71) years old, and the collected medical history included previous hypertension (80.28%), diabetes (4.23%), coronary heart disease (2.82%), Smoking history (54.93%), preoperative mean serum creatinine was 85.94umol/L, the mean serum creatinine was 113.7umol/L. 35 of 71 patients with postoperative acute kidney injury were included in the experimental group (mean age 49.97 ± 10.69 years), the remaining 36 patients were included in the control group (mean age 54.08 ± 10.48 years), The mean preoperative LVEF of all patients was 63.76% ± 6.10%. The median preoperative creatinine level was 81 mmol/L, Preoperative AST and ALT were 22U /L and 22U /L, respectively. The average intraoperative time of CPB turnaround and aortic cross clamp time was 142 min and 90 min respectively. The average infusion volume of suspended red blood cells and plasma was 600 mL and 800 ml, respectively. The average drainage was 764 ml, 1121 ml and 1406 ml 24 h, 48 h and 72 h after surgery. All patients underwent surgical treatment, and 9 patients died of after surgery. Univariate analysis showed statistically significant differences in gender (*P* = 0.013), preoperative ALT (*P* = 0.006), intraoperative transfer time (*P* = 0.008) and intraoperative suspended erythrocyte volume (*P* = 0.019) compared with the control group.

**Table 1:** Basic clinical data and univariate analysis of overweight ATAAD patients

Variable	The overall	AKI	No-AKI	*P*
Gender (male/female)	49/22	29/6	20/16	0.013
Age	52.06 ± 10.71	49.97 ± 10.69	54.08 ± 10.48	0.106
Hypertension (yes/no)	57/ 14	25/10	32/4	0.065
Diabetes (yes/no)	3/68	1/34	2/34	1.000
Coronary heart disease (yes/no)	2/69	2/33	0/36	0.239
Smoking history (yes/no)	39/32	21/14	18/18	0.397
LVEF (%)	63.76 ± 6.10	64.61 ± 5.27	62.92 ± 6.79	0.246
Preoperative Cr	81 (65, 94)	84 (67, 99)	76 (61, 91)	0.210
Preoperative AST	22 (16, 41)	24 (17, 33)	17 (15, 34)	0.069
Preoperative ALT	22 (16, 33)	30 (21, 50)	18 (14, 28)	0.006
Ascending aorta occlusion time	90 (78, 102)	93 (81, 105)	86 (73, 98)	0.102
Cardiopulmonary bypass turnaround time	142 (127, 164)	150 (131, 180)	136 (121, 152)	0.008
Intraoperative plasma infusion volume	600 (600, 600)	600 (600, 600)	600 (600, 600)	0.497
Intraoperative infusion of RBCs	800 (400, 800)	800 (613, 1232)	800 (400, 800)	0.019
Drainage 24 h after surgery	764 (598, 1003)	867 (595, 1096)	738 (602, 897)	0.128
Drainage 48 h after surgery	1121 (896, 1332)	1211 (896, 1406)	1084 (893, 1277)	0.182
Drainage 72 h after surgery	1406 (1147, 1759)	1459 (1201, 1818)	1349 (1116, 1593)	0.198

Multivariate Logistic regression analysis, gender (OR 0.243; 95% CI 0.070–0.845; *P*: 0.026), CPB turnaround time (OR 1.020; 95% CI 1.001–1.040; *P*: 0.04), intraoperative infusion of suspended red blood cells (OR 1.002; 95% CI 1.000–1.003; *P*: 0.015) was identified as an independent risk factor for postoperative AKI in overweight ATAAD patients (Table 2).

**Table 2:** Multifactor Logistics regression analysis of risk factors for postoperative AKI in overweight ATAAD patients

Variable	OR	95% CI	*P*
Gender	0.243	0.070–0.845	0.026
Cardiopulmonary bypass turnaround time	1.020	1.001–1.040	0.040
Intraoperative infusion of RBC's	1.002	1.000–1.003	0.015

## Discussion

In this study, among overweight patients with acute type A aortic dissection who developed AKI after surgery, 35 (49.30%) overweight patients developed AKI, A total of 2 patients in the experimental group underwent dialysis, while no patients in the control group underwent dialysis, and no patients required regular dialysis after discharge. 7 patients died in hospital after surgery (20%),Among them, there were 3 patients with intractable electrolyte disturbance, 2 patients with coagulation dysfunction, 1 patient with mediastinal infection, and 1 patient with suspected heparin-induced thrombocytopenia(HIT), and the overall postoperative mortality of overweight ATAAD patients was 12.68%. Multifactor Logistics regression analysis showed that male sex, CPB transit time and intraoperative infusion of suspended red blood cells were independent risk factors for postoperative AKI in overweight ATAAD patients.

In a study of aortic arch related surgery, the incidence of postoperative AKI was 43.1% [12]. Multivariate regression analysis determined that chronic kidney disease (CKD) and deep hypothermia circulation arrest > 60 min were independent risk factors for postoperative AKI. In another study of open aorta surgery, the incidence of AKI after surgery in patients with acute coronary syndrome was 70.1% [13], which was significantly higher than in our study. Lars Englberger et al. [14] found that the incidence of acute kidney injury after thoracic aortic surgery was relatively low, which was obviously inconsistent with this study and may be related to different inclusion criteria.

In this study, seven overweight ATAAD patients in the experimental group died after surgery and two patients died in the control group, with an overall mortality rate of 12.68% [15], which is similar to the previously reported mortality rate in ATAAD patients. Some large sample studies have confirmed that overweight and obese patients are more likely to develop serious complications after surgery than normal patients [10], Meanwehile, some studies have confirmed that the occurrence of postoperative AKI may increase the 10-year mortality [16]. Therefore, early identification of postoperative AKI in overweight ATAAD patients may effectively reduce patient mortality and increase long-term quality of life.

Logistic multivariate regression analysis shows that male is an independent risk factor for postoperative AKI in overweight ATAAD patients, which is consistent with previous studies [17], Marco Di Eusanio et al. examined clinical data from 1805 patients with acute type A aortic dissection with mesenteric dysperfusation [18] male gender was A predictor of postoperative mortality. Previous reports also showed that the mortality rate of female patients was higher [19–21], which may be related to the fact that the proportion of male patients (77.55%) smoking was significantly higher than that of female patients (4.55%) in the sample included in this study. Long-term smoking will further aggravate the burden of kidney and the risk of cardiovascular death [22].

The study, we evaluated the related factors in the operation in patients with overweight ATAAD postoperative AKI effect, is to identify independent risk factors of including the CPB time and intraoperative infusion quantity of suspended red blood cells, that is consistent with most of the research, with the increase of intraoperative CPB time, patients with the possibility of adverse outcome significantly increased, One study [23] showed that intraoperative circulatory pauses of less than 1 min reduced complications and the risk of bleeding from deep hypothermia. Intraoperative cryogenic extracorporeal circulation, the longer will likely affect kidney blood supply, increase renal ischemia hypoxia, cause kidney damage, plus the extracorporeal circulation leads to the activation of systemic inflammation factors, a systemic inflammatory response syndrome, thus appeared the body each viscera, the damage to the organization, and ischemia, corresponding viscera function is not complete. Secondly, the turnaround time of CPB is under the condition of deep and low temperature, and the coagulation balance of the whole body is broken, which further aggravates the burden of kidney and causes AKI [24]. In this study, suspended red blood cell infusion was an independent risk factor for postoperative AKI in overweight ATAAD patients (*P* = 0.015). This is consistent with previous research [25, 26]. It may be related to postoperative ischemia–reperfusion injury in patients undergoing cardiopulmonary bypass surgery [27]. At the same time, erythrocyte morphology and function changes in stock suspended red blood cells, including increased brittility and hemolysis reaction, can lead to postoperative renal dysfunction AKI [28]. At the same time, continuous positive fluid balance may lead to kidney damage, as increased renal volume leads to decreased glomerular filtration rate and increased load, leading to organ dysfunction and even failure [29]. K Karkouti reviewed the studies related to blood transfusion and the risk of acute kidney injury during cardiac surgery, suggesting that perioperative blood transfusion is an independent risk factor for AKI [27]. Su Wang [30] showed that transfusion of suspended red blood cells is an independent risk factor for postoperative hyperlactemia in patients with acute type A aortic dissection. Lactic acid metabolism will increase the burden on the kidney, and the kidney will further deteriorate its function while undergoing severe surgical trauma, leading to AKI.

There are many studies on postoperative AKI in Patients with ATAAD, but few studies on overweight ATAAD patients with BMI > 24. In this study, male, CPB transit time and intraoperative infusion of suspended red blood cells were found to be independent risk factors for postoperative acute kidney injury in patients with overweight acute Type A aortic dissection. It provides relevant literature support for early intervention and treatment of overweight ATAAD patients for clinicians. At the same time, the study found that postoperative AKI occurred in nearly half of overweight ATAAD patients, which reminds us to pay more attention to the prevention of postoperative AKI in overweight patients. The incidence of postoperative AKI in overweight ATAAD patients is higher, and blood Cr and urine volume of overweight patients before and after surgery should be more actively monitored. Individualized treatment for overweight patients is necessary to improve the outcome and quality of life of overweight ATAAD patients.

The current literature still lacks early laboratory indicators and biomarkers for the occurrence of postoperative AKI, and only a small part of the literature suggests that neutrophil gelatinase-related lipatin (NGAL) and cystatin C may be associated with postoperative AKI, which is only a study after cardiac surgery [31]. For ATAAD, there are no clear relevant basic studies. The conclusions drawn from this study remind clinicians to pay more attention to the prevention and management of patients with overweight ATAAD, and to avoid the occurrence of postoperative AKI as much as possible. At the same time, this study also provides a good idea for the basic research of ATAAD patients, which may help the future for drug treatment of ATAAD patients.

There are some limitations in this study. First of all, this is a retrospective study, without the confirmation of prospective studies; Secondly, the sample size of included studies was small. Furthermore, most of the patients in the study were treated with furosemide, and only preoperative and postoperative serum creatinine was selected as the inclusion criteria. Therefore, further studies with more patients are needed.

## Conclusion

In conclusion, this study suggests that men, intraoperative CPB time and intraoperative infusion quantity of suspended red blood cells for overweight patients with acute type A aortic dissection postoperative AKI independent risk factors, therefore, for patients with acute type A aortic dissection of overweight should actively to evaluate renal function, improving the prognosis of patients and improve patient quality of life, However, more patients are needed to further study the risk factors for AKI in overweight or even obese patients with acute type A aortic dissection.

## Data Availability

Contact the corresponding author for all data.

## Abbreviations

ATAAD: Acute type A aortic dissection
BMI: Body mass index
CPB: Cardiopulmonary bypass
AKI: Acute kidney injury
